# Alstrom syndrome: A rare genetic disorder and its anaesthetic significance

**DOI:** 10.4103/0019-5049.63628

**Published:** 2010

**Authors:** Akhilesh Tiwari, Disha Awasthi, Swapnil Tayal, S Ganguly

**Affiliations:** Department of Anaesthesiology and Critical Care, St. Stephens Hospital, Delhi - 110 054, India; 1 Department of Endocrinology, St. Stephens Hospital, Delhi - 110 054, India

**Keywords:** Genetic syndrome, multiorgan dysfunction, anaesthetic implications

## Abstract

Alstrom syndrome is a rare autosomal recessive disorder that was first described in 1959, by Carl Henry Alstrom, characterised by multiorgan system involvement ranging from ocular, aural, endocrinal, hepatorenal, gastrointestinal, respiratory and cardiac to the musculoskeletal system, among many others. It exposes the patient to various risks ranging from pulmonary aspiration and increased cardiac morbidity to separational anxiety, and may necessitate postoperative elective ventilation. We hereby present the successful management of one such diagnosed case in a 12-year-old boy, who presented to us for incision and drainage of an abscess present over the nape of his neck, along with foreign body removal from his right ear.

## INTRODUCTION

Alstrom syndrome is a rare autosomal recessive disorder comprising of progressive vision loss, progressive sensorineural hearing loss, morbid obesity, male hypogonadism, insulin-resistant diabetes mellitus, renal failure and dilated cardiomyopathy.[1] It is characterised by multiorgan dysfunction, sharing several features with the common metabolic syndrome namely obesity, hyperinsulinemia and hypertriglyceridemia.[2] This syndrome was first described by Carl-Henry Alstrom, in Sweden, in 1959. We present here the management of a case of this syndrome, which poses several challenges to an anaesthesiologist.

## CASE REPORT

**Figure 1:**
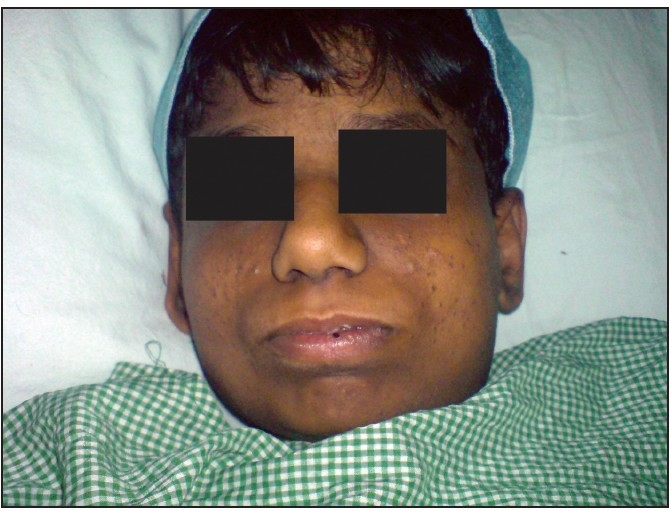
Alstrom syndrome

A 12-year-old patient, weighing 52 kg, and a known case of Alstrom syndrome presented to an ENT specialist with a foreign body in the right ear, abscess over the nape of the neck and recurrent episodes of acute bronchitis [Figure 1]. On detailed history the patient had gastro-oesophageal reflux disorder (GERD) grade III with gastropathy, hiatus hernia, hypothyroidism, acanthosis nigricans, hepatosplenomegaly and pancytopenia. He had a history of sudden severe sensorineural hearing loss which improved with a short course of steroid. The child also gave a history of photophobia with nystagmus along with 100% visual disability. The patient also had secondary metabolic myopathy which involved the proximal muscles and was more severe in the upper limbs. On physical examination, the patient was pale and had a barrel-shaped chest with kyphosis. Air entry was decreased in the base of the lung, on the right side, with diffuse rhonchi present all over the lung fields. The liver was palpable four centimeters and the spleen was palpable six centimeters below the costal margin. A chest X-ray revealed opacity on the right side base of the lung. The patient had deranged liver function and normal kidney function, with platelet counts varying from 30,000 to 40,000 per microlitre in spite of repeated platelet transfusion. His haemoglobin was 6.7 gm%. His 2-D ECHO was normal. Endoscopy revealed a small oesophageal varix with erosive gastroduodenopathy, GERD and hiatus hernia. The patient was started on injection augmentin and injection monocef, and was nebulised with budecort, following which there was significant improvement in his chest condition and he was maintaining SpO_2_ of 100% on room air. The only long-term treatment he was on was Tab. Pantoprazole 40 mg OD PO and Tab Thyroxine 50 *µ*g OD PO.

The patient was posted for foreign body removal from right ear, along with incision and drainage. After a detailed pre-anaesthetic check up, the patient was classified under MPC II and ASA III. The patient was advised steam inhalation and nebulisation with a combination of levosalbutamol sulphate and Ipratropium brominde. The patient was given Tab. Midazolam 7.5 mg one hour before the surgery, along with aspiration prophylaxis, and once this was received in the preoperative room, the patient was again nebulised and then shifted to the OR. However, the patient was very anxious when he was brought into the operating room. After attaching all the routine monitors, 1 mg Midazolam was administered intravenously, analgesia given with Injection fentanyl 75 *µ*g and Propofol was given on a titrated basis, the total dose being 80 mg. Awake intubation was done with a cuffed tracheal tube of 6.5 mm, as adequate prophylaxis was given against aspiration, and the patient was later shifted to the left lateral position, and maintained on a combination of oxygen, nitrous oxide and volatile anaesthetic sevoflurane. Relaxation was achieved by using injection Atracurium 15 mg initially and 5 mg was repeated after 20 minutes. The intraoperative course was uneventful, and the patient was transfused with two units of platelets preoperatively, and one unit of crystalloid and one unit of platelets intraoperatively. The whole procedure lasted for 35 minutes and was uneventful, without any unwanted bleeding. The patient was extubated when adequately reversed and spontaneously breathing with intact airway reflexes.

## DISCUSSION

The aim of presenting this case report is to highlight this rare syndrome and it is worthwhile to mention that there are only 266 recorded cases in the literature so far. Alstrom syndrome is a rare autosomal recessive multisystemic disorder characterised by retinal degeneration, obesity, progressive sensorineural hearing loss, non-insulin-dependent diabetes mellitus, renal failure and heart failure. Mental retardation is usually absent and the extremities are normal.[3]

Therefore, it is imperative at this juncture, to highlight the various anaesthetic implications of the Alstrom syndrome. Presence of progressive vision loss and progressive sensorineural hearing loss, as seen in this patient, lead to severe situational disturbances and anxiety. This patient also had GERD grade III and hiatus hernia, which predisposed them to aspiration, making aspiration prophylaxis a must. Presence of insulin-resistant diabetes mellitus leads to Autonomic Dysregulation, which needs to be kept in mind. Presence of metabolic myopathy posed an additional problem with respect to administration of Succinylcholine. The patients of Alstrom syndrome also have dilated cardiomyopathy, which requires the maintenance of cardiac output to a near normal level, along with the mandatory monitoring of blood pressure either by the invasive or non-invasive method. However, fortunately there was no cardiac involvement seen in our patient.

The presence of hepatic dysfunction and renal failure makes the judicious selection of an anaesthetic agent a must. The presence of pancytopenia may pose an additional risk during regional anaesthesia. Transportation and shifting of the patient at every stage requires gentle handling due to the presence of scoliosis, kyphosis, metabolic myopathy and muscle dystonia. Other features associated with Alstrom syndrome include alopecia, short stature, hyperostosis frontalis interna, muscle dystonia, advanced bone age and subcapsular cataract.[3]

Also observed is growth hormone deficiency and hyperuricemia.[3] Progressive vision loss has been attributed to cone rod retinal dystrophy leading to juvenile blindness.[4] Most patients demonstrate normal intelligence although some reports indicate delayed psychomotor and intellectual development; also associated is pulmonary fibrosis and restrictive lung disease.[4] In the final stage, the disease affected individual exhibits progressive chronic nephropathy, with eventual kidney failure. Diagnosis of AS should be considered in infantile cone and rod retinal dystrophy particularly if the weight is above the ninetieth percentile or if there is infantile cardiomyopathy.[5] The most frequent cause of death includes hepatic dysfunction and CHF, secondary to dilated cardiomyopathy. Life span rarely exceeds 40 years.[4]

Mutation of the ALMS1 gene located in chromosome 2p has been found to be causative.[2,3] Age of onset, and severity vary greatly among and within families. The ALMS protein is also found at the base of the cilia. Thus, Alstrom syndrome is a ciliopathy, associated with other known ciliopathies that include primary ciliary dyskinesia, Bardet Biedl syndrome, polycystic kidney disease, nephronophthiasis, Meckel-Gruber syndrome and some form of retinal degeneration.[6] There is no specific therapy for the Alstrom syndrome, but early diagnosis and intervention can moderate the progression of the disease phenotype and improve the longevity and quality of life of the patient, and the aim of presentation of this case is to put forward important anaesthetic implications of this rare syndrome, as it may present in major surgeries like splenectomy as well.

## CONCLUSION

Alstrom syndrome is one of the rarest genetic syndromes having an autosomal recessive mode of inheritance, and it is extremely unlikely that such a case would be encountered on a routine basis in the operating room. We hereby report the successful management of such a case which poses numerous anaesthetic challenges. Detailed preoperative evaluation and meticulous planning of anaesthesia go a long way in the successful management of such a case.
